# The Association between Dietary Inflammatory Index (DII) and Cancer Risk in Korea: A Prospective Cohort Study within the KoGES-HEXA Study

**DOI:** 10.3390/nu11112560

**Published:** 2019-10-23

**Authors:** Injeong Ryu, Minji Kwon, Cheongmin Sohn, Nitin Shivappa, James R. Hébert, Woori Na, Mi Kyung Kim

**Affiliations:** 1 Department of Food and Nutrition, Yonsei University, Seoul 03722, Korea; dlswjd5158@gmail.com; 2 Division of Cancer Epidemiology and Management, National Cancer Center, Goyang-si, Gyeonggi-do 10408, Korea; 74433@ncc.re.kr; 3 Department of Food and Nutrition, Wonkwang University, Iksan-si, Jeollabuk-do 54538, Korea; ccha@wku.ac.kr (C.S.); nawoori6@gmail.com (W.N.); 4 Cancer Prevention and Control Program, University of South Carolina, Columbia, SC 29208, USA; SHIVAPPA@mailbox.sc.edu (N.S.); JHEBERT@mailbox.sc.edu (J.R.H.); 5 Department of Epidemiology and Biostatistics, Arnold School of Public Health, University of South Carolina, Columbia, SC 29208, USA; 6 Connecting Health Innovations LLC, Columbia, SC 29201, USA

**Keywords:** cancer, inflammatory mediator, diet, antioxidant, epidemiology, dietary inflammatory index

## Abstract

Several epidemiological studies have shown that there are consistently positive associations between dietary inflammatory index (DII^®^) scores and cancer incidence in Western populations. However, few DII-cancer studies have been conducted in East Asian populations. In a large cohort representative of the general Korean population, we investigated whether the DII is associated with overall cancer risk. A total of 163,660 participants (56,781 males and 106,879 females) had evaluable data for analyses. This follow-up study was carried out over the course of 7.9 years. DII scores were calculated based on Semi-Quantitative Food-Frequency Questionnaire (SQ-FFQ) data for 106 food items. Cancers were self-reported based on notification by the participants’ medical doctors. Multivariable Cox proportional hazard regression was used to estimate hazard ratios (HRs) with 95% confidence intervals (CIs). After the follow-up, 1643 incident cases of cancer (520 males and 1123 females) had developed. In a fully adjusted model, women in the highest DII quintile showed a 44% increased risk of getting cancer (HR_Q5vsQ1_ = 1.44; 95% CI = 1.14–1.82; *p*-trend = 0.0006), while men showed no apparent association (HR_Q5vsQ1_ = 0.80; 95% CI = 0.58–1.10). These results indicate that in Korean women, a more pro-inflammatory diet is associated with a higher risk of incident cancer.

## 1. Introduction

Cancer is the generic term for a group of diseases that arises from abnormal cell growth, which can lead to metastasis in a multistage process. According to the World Health Organization (WHO), cancer is the second leading cause of death worldwide, responsible for 9.6 million deaths in 2018, notably causing 70% of deaths in developing countries [1]. In South Korea, cancer is the first leading cause of death: The number of deaths caused by cancer in 2018 was 86,281, 28.9% of the total incidence of death [2]. Development of cancer is influenced by genetic, environmental, and lifestyle factors. The main risk factors are tobacco, alcohol, unhealthy diet, and physical inactivity, while other factors include radiation, stress, environmental pollutants, and genetic defects [1,3]. Cancer onset inflicts physical and mental pain on patients and their families and puts them under financial strain. Furthermore, from the view of society, it leads to economic losses due to decreased human resources and productivity, while increasing national healthcare expenditures [4]. Hence, it is important to map out strategies for cancer prevention in order to reduce financial burden and suffering on individuals and for the nation as a whole.

Inflammation is part of the innate immune reaction that responds to tissue damage induced by pathogens, damaged cells, or irritants [5,6,7]. When inflammation occurs, activated macrophages and lymphocytes secrete inflammatory mediators, amplifying immune response [8]. There is robust evidence that several inflammatory mediators such as tumor necrosis factor (TNF-α), interleukin (IL)-6, IL-10, transforming growth factor β (TGF-β), and high-sensitivity c-reactive protein (hs-CRP) are critical components of oxidative DNA damage, triggering malignant tumor progression [6,9,10]. Meanwhile, previous studies have verified that diet can modulate levels of inflammatory mediators [11,12,13] and cancer development [14,15]. The degree to which inflammation occurs as a result of diet can be determined via the dietary inflammatory index (DII^®^), a score that measures the inflammatory potential of food items based on dietary patterns within any study population [16].

Several studies have shown that higher DII score is associated with elevated levels of inflammatory mediators, which include TNF-α, IL-6 and hs-CRP, indicating strong link between DII and cancer [8,17,18,19,20,21]. Many epidemiological studies have also contributed towards growing evidence for associations between DII and specific cancers [22,23,24,25,26,27,28,29,30]. On the other hand, several other such studies have shown only ambiguous associations between DII and cancers [31,32]. Notwithstanding these conflicting results, a majority of studies, including meta-analyses, have concluded that there are consistent positive associations between DII and cancer incidence across cancer types, populations, and study designs [33,34,35,36,37,38,39,40,41,42,43,44]. However, most of the relevant studies have been conducted with Western populations and have targeted specific cancers. As far as we know, there have been few pertinent studies thus far on East Asian populations [45,46,47,48,49,50]. In the present large cohort study representative of the Korean population, which entails 7.9 years of follow-up, we tested the hypothesis that a higher DII score is associated with higher risk of cancer.

## 2. Materials and Methods

### 2.1. Data Collection and Subject Recruitment

The data used in this study were collected from the cohort of the KOREA GENOME and Epidemiology Study (KoGES), including the KoGES_Ansan and Ansung study, the KoGES_cardiovascular disease association study (CAVAS), and the KoGES_health examinee study (HEXA). In order to gather participants’ genetic, environmental, and lifestyle information affecting cancer expression, candidate subjects based on the Korean population, both males and females aged ≥40 years, had been recruited from the National Health Examinee Registry. Data representative of that cohort from the KoGES-HEXA study were used in the present study to examine the association between DII and cancer expression. Detailed information on the KoGES can be found elsewhere [51].

**Figure 1:**
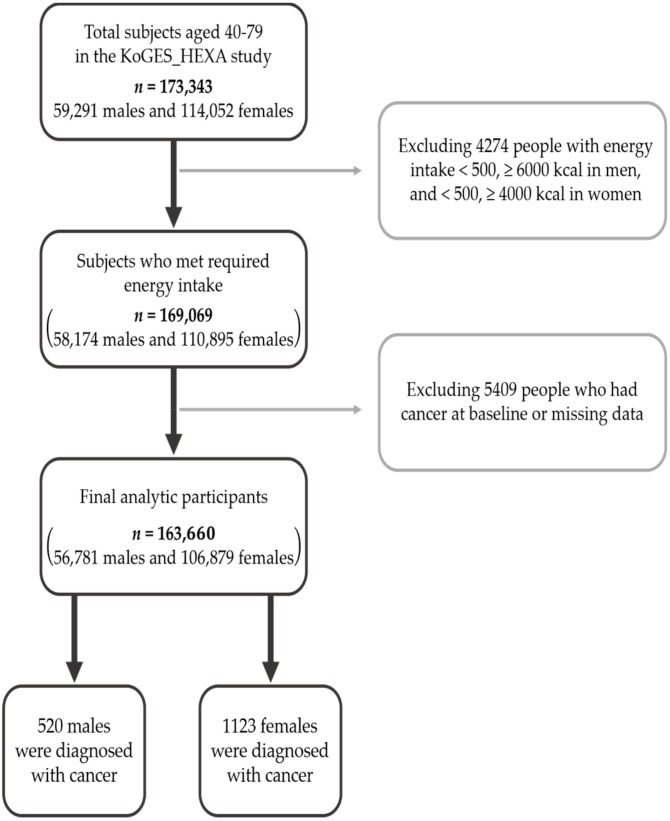
Flow chart of analytical samples in KoGES_HEXA Study.

The participants were asked to respond to the multiple-choice survey voluntarily. The 173,343 participants (59,291 males and 114,052 females) were enrolled from 38 health examination centers and hospitals located in eight regions in Korea between 2004 and 2013 and were asked to attend a follow-up study conducted over the course of the 7.9 years (2007–2016). Among this original population, 4274 people who had insufficient energy intake (males <500 kcal or ≥6000 kcal; females <500 kcal or ≥4000 kcal) and 5409 people who had cancer already at the baseline or missing data were excluded. Thus, the valid subjects numbered 163,660 (56,781 males and 106,879 females). After the 7.9-year follow-up study, the total cancer incidence was 1643 (520 cases in males and 1123 cases in females), and identification of cancer expression was self-reported after diagnosis by a medical doctor (Figure 1). All of the subjects provided written consent prior to this study, which was approved by the Institutional Review Board of the National Cancer Center in Korea and followed all of the relevant guidelines and regulations (IRB No. NCC2018-0164).

### 2.2. DII Calculation

Dietary food intake was measured with a Semi-Quantitative Food Frequency Questionnaire (SQ-FFQ). The validity of the SQ-FFQ was proven in a previous study [52]. Dietary intake was measured once, at the beginning of the study, regarding the past year of intake. The study participants reported the daily intake amounts (g), average portion sizes and serving frequencies of 106 foods. The portion size was divided into three levels: half a serving, a standard serving, and three-half servings, and frequencies were categorized into nine levels, from “almost never” to “more than three times a day”. Total calories and nutrient intakes were calculated with a Korean standard food composition table [53].

For the updated version of DII, 1943 articles were reviewed and scored. Forty-five food parameters, including foods, nutrients, and other bioactive compounds, were identified according to their capacity for changing the levels of specific inflammatory markers (i.e., hs-CRP, IL-1β, IL-4, IL-6, IL-10, and TNF-α). Regionally representative data sets based on diet surveys from 11 countries were collectively used as comparative standards for each of the 45 parameters.

To calculate the DII scores, the intake scores of the above-noted data sets were applied. A paper on DII methodology provides more detailed information [16]. To summarize, Z scores were generated by subtracting a standard mean, as derived from the world data sets for each food parameter, from the actual scores and dividing it by its standard deviation. These Z scores were converted into percentile ranks in order to minimize the effects of outliers or right-skewing. These values were doubled, and 1 was subtracted, to make the distribution symmetric relative to 0. The resulting values were then multiplied by the corresponding inflammatory score for each food parameter, and all were summed to obtain the overall DII score.

In this study, the following 37 food parameters were adopted to calculate DII: anthocyanidins, green and black tea, Zn, Mg, Fe, Se, vitamins (A, B_6_, B_12_, C, D, E), thiamin, MUFA, PUFA, niacin, garlic, onion, folic acid, fiber, protein, total fat, saturated fatty acids, trans fat, n-3 fatty acid, n-6 fatty acid, carbohydrate, cholesterol, caffeine, carotene, riboflavin, isoflavone, flavanone, flavonol, flavones, flavan-3-ol, and calories. From among these 37 food parameters, the pro-inflammatory parameters include vitamin B_12_, total fat, protein, saturated fatty acids, trans fat, n-6 fatty acid, carbohydrate, cholesterol and calories, and anti-inflammatory parameters include anthocyanidins, green and black tea, Zn, Mg, Fe, Se, vitamins (A, B_6,_ C, D, E), thiamin, MUFA, PUFA, niacin, garlic, onion, folic acid, fiber, n-3 fatty acid, caffeine, carotene, riboflavin, isoflavones, flavanone, flavonol, flavones, and flavan-3-ol.

### 2.3. Covariates

In the present study, 12 covariates, representing a questionnaire on personal and family medical history, physical and mental health, lifestyle, and FFQ, were selected as potential moderator variables. These included age, BMI, and energy for continuous variables and gender, marital status, educational level, income, smoking, drinking, physical activity, menopausal status, and family history of cancer for categorical variables. Gender was sorted by biological sex: male and female. Marital status was divided into two categories: married and single or divorced or widowed. Educational level was divided into three levels: <Middle school and Middle school to College and ≥College. Monthly income was recorded based on 10,000 won as the unit and was divided into four levels: <100, 100~200, 200~300, and ≥300. With regard to smoking, subjects who had been smoking more than 400 cigarettes at the time of the survey were classified as “current”, who had smoked more than 400 cigarettes but quit smoking as “past”, and who had not smoked more than 400 cigarettes as “never”. As for drinking, those who had been drinking at the time of the survey were classified as “current”, those who had been drinking but quit as “past”, and those who had never drunk as “never”. Menopausal status was sorted into post-menopause and pre- or peri-menopause, and it was determined by whether participants experienced menstrual cycles within the past one year. Regular physical activity was defined as exercising regularly enough to sweat. Those who fulfilled the criteria were considered as doing “regular” physical activity, while those who did not fulfill the criteria were considered as doing “irregular” physical activity. Family history of cancer was classified into “Yes” and “No”.

### 2.4. Statistical Analysis

The study subjects were classified into five groups (quintiles) based on their DII scores. DII quintiles were divided based on a no-incidence group. Continuous variables were presented as means with standard deviation, and categorical variables were presented as frequency numbers with percentages. To calculate the *p* values for trends, the Jonckheere–Terpstra test and the Mantel–Haenszel Chi-square test were applied to the continuous variables and categorical variables, respectively. The Cox proportional hazard model was used to find any association between the DII quintiles and cancer incidence. The fully adjusted model was adjusted for age and energy as continuous variables and sex (for the total), marital status, education, smoking, drinking, physical activity, and family history of cancer as categorical variables. The HRs were calculated with 95% confidence intervals. To confirm the assumption of proportional risk, the models including time-dependent covariates were evaluated. To determine the effects of the DII components on cancer risk, the contents of the food parameters were adjusted for energy and divided into quintiles, after which the multivariable proportional hazard model was re-applied. *p* values for trends were calculated for both continuous and categorical DII and DII components. Additionally, the *p* interaction for gender and categorical DII in the fully adjusted Cox model was calculated. *p* < 0.05 was deemed statistically significant. All of the statistical tests were performed using SAS program version 9.4 (SAS Institute, Cary, NC, USA).

## 3. Results

The demographic characteristics of 163,660 subjects according to DII quintiles are presented in Table 1. The range of the highest DII quintile is 2.1973 to 7.1056 (SD = 0.82) and the lowest DII quintile is −9.1296 to −0.9589 (SD = 1.08). As DII increased, the mean age of the subjects increased, while the mean BMI and mean energy intake have tended to decrease. The proportion of females decreased in higher-DII quintiles. The proportion of participants who were married decreased as DII increased. The income level and educational level also decreased as DII increased. To put it concretely, in higher (i.e., more pro-inflammatory) DII quintiles, there were more people earning less than 1,000,000 won a month but fewer people earning more than 3,000,000 won a month. There also were more people who had not finished middle school but fewer people who had more than a bachelor’s degree. The proportion of “current” smokers showed a tendency to increase and that of “never” smokers to decrease, as DII increased, while the proportions of “current” drinkers decreased and “never” drinkers increased. The percentage of women of post-menopausal status was higher as DII increased. By contrast, the percentage of people who exercised regularly was lower as DII increased. Family history of cancer did not show any remarkable tendency across DII quintiles. To sum up, as the DII increased, mean age, proportion of males, proportion of smokers, and proportion of post-menopausal status increased, while mean BMI, mean energy intake, proportion of married status, educational level, income level, proportion of drinker, and proportion of people who exercise regularly decreased. All of the *p* values were less than 0.0001.

After carrying out 7.9-years follow-up on 163,660 subjects, 1643 cases of cancer (520 males and 1123 females) were detected. The most common cancer in women was breast cancer (239 cases), and the most common cancer in men was gastric cancer (137 cases). The rate of overall cancer development was 14% higher for women (1.05%) than for men (0.92%). To identify whether DII is associated with cancer risk, a proportional hazard model was applied, and nine possible confounding variables listed in Table 1 were used to generate a fully adjusted hazard model.

The results stratified by gender are presented in Table 2. As can be seen, a statistically significant result was observed only among females. In the fully adjusted hazard model, women with the highest DII range (=Q5) had a 44%-higher risk of getting cancer relative to the reference (=Q1) (HR = 1.44; 95% CI = 1.14–1.82). The *p* trend also showed strong evidence in its significance (categorical DII = 0.0022; continuous DII = 0.0006). We also observed a near significant association for overall analyses. (HR = 1.20; 95% CI = 0.99–1.45). However, there were no statistically significant associations between DII and cancer incidence among males (HR = 0.80; 95% CI = 0.58–1.10). The *p* interaction between gender (male/female) and categorical DII in the fully adjusted Cox model was 0.0594.

In order to examine which of the 37 food parameters applied to DII calculation facilitate or reduce the risk of cancer, the proportional hazard model was applied again.

As shown in Table 3, statistically significant results for DII components were observed in women. Among the 37 food parameters, seven (isoflavone, flavanone, flavonol, flavan-3-ol, green and black tea, riboflavin, and iron) were found to reduce cancer incidence. Compared with the reference (=Q1), the hazard ratio (HR) of the highest DII components’ contents (=Q5) showed that isoflavone reduces the incidence of cancer in women by 22% (HR = 0.78; 95% Cl = 0.64–0.95), flavanone by 19% (HR = 0.81; 95% CI = 0.67–0.98), flavonol by 34% (HR = 0.66; 95% CI = 0.54−0.82), flavan-3-ol by 32% (HR = 0.68; 95% CI = 0.55–0.84), green and black tea by 35% (HR = 0.65; 95% CI = 0.54–0.77), riboflavin by 19% (HR = 0.81; 95% CI = 0.67–0.99), and iron by 20% (HR = 0.80; 95% CI = 0.66–0.97). However, in terms of *p* trends, six components (isoflavone, flavonol, flavan-3-ol, green and black tea, riboflavin, and iron) showed strong evidence of their significance, while flavanone showed only weak evidence (categorical component = 0.3148; continuous component = 0.1618).

## 4. Discussion

In this large Korean cohort study, we investigated the association between inflammatory potential of diet, referred to as DII, and the risk of cancer development. We determined that a pro-inflammatory diet (the highest DII scores) increased the risk of cancer in women. After a 7.9-year follow-up, the result obtained for women showed a 44% higher risk of getting cancer in the most pro-inflammatory DII quintile compared with the lowest DII quintile (representing the most anti-inflammatory diet) after multivariable adjustment. Moreover, a similarly positive association was found between energy-adjusted DII (E-DII) and risk of cancer among women (HR_Q5vsQ1_ = 1.27 (1.04–1.56)). However, no significant result was observed for men. These results are partially consistent with our hypothesis that higher DII is associated with higher risk of cancer in the Korean population.

There have been many studies examining the association between DII and cancer incidence. Most case-control studies and cohort studies have shown positive associations between DII and cancer incidence [23,24,25,26,27,28,29,30]. In addition, there have now been over 10 meta-analyses that have all shown consistently positive results [33,34,35,36,37,38,39,40,41,42,43,44]. On the other hand, some studies have reported statistically non-significant associations between DII and cancer incidence. In a cohort-study involving ten screening centers across the United States, no statistically significant association was observed between DII and pancreatic cancer (HR_Q5vsQ1_ = 1.31 (0.83–2.08)) [31]. Moreover, in a case-control study in Mexico, likewise, there was no significant association between prostate cancer risk and E-DII (OR_T3vsT1_ = 1.18 (0.85–1.63)) [32].

However, most of the aforementioned studies targeted specific cancers that appear usually in only one gender, such as breast cancer, cervical cancer, and prostate cancer, or analyzed without stratification of gender. Among the investigations on the association between DII and cancer incidence, only a few have shown gender difference in their results. In these studies, most of them found significant results for men but not for women. According to a prospective cohort study that used data from the Västerbotten Intervention Programme, researchers observed a statistically significant result, which was that an anti-inflammatory diet helped to reduce the risk of lung cancer (men; HR_T3vsT1_ = 0.81 (0.66–0.99), women; HR_T3vsT1_ = 0.89 (0.74–1.08)) and gastric cancer (men; HR _T3vsT1_=0.73 (0.53–0.99), women; HR_T3vsT1_ = 0.97 (0.70–1.34)) only in men [54]. Meanwhile, a Japanese study on the association between DII and hs-CRP (which is used as an index of cancer screening) suggested that DII is correlated with elevated hs-CRP level in Japanese men but not in women (men; OR_CRP>0.3mg/dLvs≤0.3mg/dL_ = 1.17 (1.02–1.35), women; OR_CRP>0.3mg/dLvs≤0.3mg/dL_ = 0.99 (0.79–1.24)) [55]. In a case-control study from Iran, a significant association between DII and colorectal cancer was found, once again, only in men (men; OR_DII>-0.23vs≤-0.23_ = 33.95 (3.72–309.44), women; OR_DII>-0.23vs≤-0.23_ = 0.60 (0.22–1.61)) [56].

In our study, however, there was a positive association between DII and cancer incidence not for men but only for women. To the best of our knowledge, there have been only two study results coinciding with the current ones. Case-control studies on Korean populations showed a positive association between DII and gastric cancer (men; OR_T3vsT1_ = 1.31 (0.84–2.05), women; OR_T3vsT1_ = 2.98 (1.68–5.30)) [57] and proximal colon cancer (men; OR_T3vsT1_ = 1.51 (0.89–2.57), women; OR_T3vsT1_ = 2.23 (1.02–4.89)) only in women [58], suggesting that the associations between DII and cancer incidence are stronger in women than in men in this population. 

Although studies that support our results are not sufficiently numerous, previous studies investigating gender differences in immune response provide possible explanations. Due to differences of hormonal status and rare genes on the X chromosome, inflammation-related genes of females can be over expressed, resulting in poorer prognoses for females when they suffer from chronic inflammatory diseases such as rheumatoid arthritis, inflammatory bowel disease, and other autoimmune diseases [59,60,61]. Additionally, in the process of inflammation, hs-CRP levels, the erythrocyte sedimentation rate, and neutrophil counts, which are biomarkers used to identify cancer incidence, are higher for females than for males [61,62,63]. These results substantiate the contention that chronic inflammatory disease is predominant in women. However, some researchers have posited conflicting mechanisms with other chronic inflammatory disease in the case of cancer, due to differences in physiology, regulation of gene expression and epigenetic mechanisms, and due also to the fact that, as for most cancers, males generally have higher susceptibility than females [64]. As noted above, evidence in the form of epidemiological results is also accumulating [54,55,56]. The obscure effect of the DII in men in this study may be related to the simple fact that men, in general, have more pro-inflammatory diets than women. In this study, men had a higher mean DII score (0.684; SD = 1.96) than did women (0.610; SD = 1.93). Moreover, there are statistically significant differences in DII scores between men and women (Wilcoxon rank-sum test, *p* < 0.0001). It is possible that the DII distribution for men was so skewed to the pro-inflammatory diet (higher DII) that it could not fully reflect the inflammatory potential of diet. Since not only there are no tangible reasons for gender differences in associations between DII and cancer incidence but also the results of our study contradict those of previous studies, further investigations with larger sample sizes and sufficient statistical power are needed in order to determine whether there are any gender differences in the associations between DII and cancer incidence.

The present study also confirmed that among the 37 DII components, seven (isoflavone, flavanone, flavonol, flavan-3-ol, riboflavin, green and black tea, and iron), known as antioxidants, help reduce cancer risk in women. Several mechanisms have been proposed to explain the effects of antioxidants in alleviating inflammation. In the inflammatory response, an oxidative environment is formed, since leukocytes and mast cells release reactive oxygen species (ROS), while inflammatory cells generate inflammatory mediators in the damage regions [65,66,67]. Under the healthy condition, the human body can balance between ROS and antioxidant enzymes; however, if the body’s equilibrium is disturbed, oxidative stress, which might trigger carcinogenesis in turn, can be incurred [68,69]. Antioxidant supplements help to avoid oxidative stress by balancing ROS. Thereby, they prevent vital cellular components such as DNA, proteins, and membrane lipids from being damaged, which normally would lead to cell death [67,70]. In particular, flavonoids, known as typical antioxidants, exert a protective role in tumor development by inhibiting cancer cell growth and proliferation with concomitant induction of apoptosis in the absence of cytotoxicity [71]. Moreover, riboflavin (vitamin B2), as a component of the glutathione redox cycle, has antioxidant properties [72], and iron also protects cells from the effects of free radicals, specifically in its role as a supplier of effective reducing agents, ferric ions (Fe^3+^) [73]. In line with the above mechanisms, we found that riboflavin, iron, three subtypes of flavonoids (i.e., isoflavone, flavonol and flavan-3-ol) and green and black tea, which is rich in polyphenols, including flavonoids and phenolic acid, have strong effects in reducing cancer incidence. However, flavanone shows weak evidence of any significance, and flavone and anthocyanidin, two other subtypes of flavonoids, along with onion, which contain much quercetin, a type of flavonol, show non-significant results. Such results, moreover, have been observed only in women.

Another interesting finding is that the proportion of “current” drinkers was higher in the lower DII quintiles. This is consistent with a previous study on the association between alcohol consumption and concentrations of inflammatory biomarkers. Daily alcohol intake showed an apparent U-shaped association with hs-CRP and fibrinogen. In other words, moderate alcohol consumption has negative correlations with elevated inflammatory biomarkers, appearing to have an anti-inflammatory effect [74,75]. However, given that "moderate” alcohol consumption may vary by individual, and given also that there are heterogeneous epidemiological results indicating that even moderate alcohol consumption increases cancer incidence [76], further epidemiological studies involving subjects of diverse demographic characteristics are needed in order to determine what properly represents a “moderate” amount of alcohol. Also required are studies on the associations among inflammation, cancer incidence, and moderate alcohol consumption.

In addition, we saw caloric intake decreased in higher-DII quintiles, which is contrary to the common notion that calories are pro-inflammatory components. Underlying our observations in light of all of the many studies which have been conducted to date using the DII are two countervailing effects. The first is a positive correlation between energy intake and nutrient intake, resulting from a tendency to eat more of everything as one increases energy intake. The other is “healthy eater” effect (e.g., health-conscious people prefer choosing nutrient-dense and energy-sparse foods) [77]. In this study, we anticipated the first effect was more dominant than the “healthy eater” effect. The subjects with lower DII followed a more anti-inflammatory diet such as fruits and vegetables, which are rich in vital micronutrients and bio-active compounds as well as macronutrients. Since there is a positive correlation between energy intake and nutrient intake, consuming more fruits and vegetables contributed to greater caloric intake. Hence, in lower DII quintile, we saw higher values of not only pro-inflammatory components like energy and macronutrients but also vital micronutrients and other bioactive compounds considered to be anti-inflammatory components.

There are several limitations to the present study. First, selection bias might have been incurred. The subjects had been recruited from 38 health examination centers and hospitals located in urban areas of Korea, and only those willing to participate were enrolled; consequently, the number of women who consented to the study was higher than that of men; they might not be entirely representative of the Korean population. This type of screening bias occurs in many prospective cohort studies [78]. Second, cancer diagnosis was self-reported, and as such, errors in the numbers of cancer incidences might have been incurred. Third, among the 45 food parameters of DII, we used data on only 37, excluding the remaining eight. Although this can be considered to be a study limitation, it was perhaps not a major factor having a significant impact on the outcome, because those eight components are not frequently consumed. Moreover, whereas we identified the effects of the overall inflammatory mediators on cancer incidence through DII, we did not observe the effects of individual inflammatory markers such as hs-CRP, IL-1β, IL-4, IL-6, IL-10 or (TNF)-α. The lack of information on associations between DII and specific cancers might be another limitation, since we targeted all cancers. In spite of the above limitations, our study has strengths. It is a prospective cohort study with a large sample size and a long-term follow-up. Hence, relative to case-control studies, it reduces the chance of recall bias and provides reliable results. Above all, the present study is important in terms of its provision of accumulated evidence of a positive association between DII and cancer risk. We expect that the present study will contribute to the enhancement of public health by emphasizing the importance of a healthy diet and also by raising public awareness of the dangers of a pro-inflammatory diet.

## 5. Conclusions

Higher DII scores were associated with higher risk of cancer incidence in Korean women. More epidemiological studies, particularly those on the association between DII and cancer for various world populations, are needed.

## Figures and Tables

**Table 1:** Selected variables at baseline according to Dietary Inflammatory Index (DII), KoGES 2004–2013.

Variables ^b^ (Mean (SD) or *n* (%))	Quintiles of Dietary Inflammatory Index (DII) ^a^					*p* Value ^c^
	Q1	Q2	Q3	Q4	Q5	
	*n* = 32,757	*n* = 32,721	*n* = 32,757	*n* = 32,733	*n* = 32692	
	−9.1296–−0.9589	–0.9588–0.4180	0.4180–1.3036	1.3036–2.1973	2.1973–7.1056	
Age (years)	52.2 (8.0)	52.3 (8.2)	52.7 (8.2)	53.3 (8.5)	54.6 (8.7)	<0.0001
BMI (kg/m^2^)	24.1 (2.9)	24.0 (2.9)	24.0 (2.9)	23.8 (2.9)	23.8 (3.0)	<0.0001
Energy (kcal/day)	2266.0 (580)	1901.5 (425)	1669.8 (373)	1496.3 (388)	1407.7 (366)	<0.0001
Gender						
Male	11,002 (33.6)	11,419 (34.9)	11,513 (35.2)	11,207 (34.2)	11,640 (35.6)	<0.0001
Female	21,755 (66.4)	21,302 (65.1)	21,244 (64.9)	21,526 (65.8)	21,052 (64.4)	
Marital status						
Married	29,345 (90.2)	29,218 (89.9)	28,955 (88.8)	28,279 (86.9)	27,474 (84.5)	<0.0001
Single/Divorced/Widowed	3198 (9.8)	3298 (10.1)	3638 (11.2)	4274 (13.1)	5029 (15.5)	
Education level						
<Middle school	3915 (12.1)	4404 (13.7)	5241 (16.2)	6359 (19.7)	8631 (26.8)	<0.0001
Middle school~College	19,132 (59.2)	18,885 (58.5)	18,684 (57.8)	18,518 (57.4)	17,795 (55.2)	
≥College	9271 (28.7)	8983 (27.8)	8423 (26.0)	7406 (22.9)	5792 (18.0)	
Monthly income (10,000 ￦)						
<100	2178 (8.1)	2343 (8.5)	2851 (10.4)	3670 (13.3)	4982 (17.8)	<0.0001
100~200	4843 (18.1)	5167 (18.8)	5472 (19.9)	5954 (21.6)	6811 (24.4)	
200~300	6175 (23.0)	6580 (23.9)	6298 (22.9)	6110 (22.2)	5946 (21.3)	
≥300	13,609 (50.8)	13,426 (48.8)	12,909 (46.9)	11,803 (42.9)	10,232 (36.6)	
Smoking						
Never	24,091 (73.9)	23,694 (72.7)	23,761 (72.8)	23,904 (73.3)	22,921 (70.3)	<0.0001
Past	4567 (14.0)	4957 (15.2)	4953 (15.2)	4745 (14.5)	4758 (14.6)	
Current	3940 (12.1)	3941 (12.1)	3940 (12.1)	3977 (12.2)	4919 (15.1)	
Drinking						
Never	16,051 (49.2)	15,902 (48.8)	16,257 (49.8)	16,727 (51.3)	17,203 (52.8)	<0.0001
Past	1250 (3.8)	1157 (3.6)	1131 (3.5)	1303 (4.0)	1288 (4.0)	
Current	15,319 (47.0)	15,541 (47.7)	15,284 (46.8)	14,606 (44.8)	14,114 (43.3)	
Physical activity ^d^						
Irregular	11,002 (33.6)	11,419 (34.9)	11,513 (35.2)	11,207 (34.2)	11,640 (35.6)	<0.0001
Regular	21,755 (66.4)	21,302 (65.1)	21,244 (64.9)	21,526 (65.8)	21,052 (64.4)	
Menopausal status						
Post-menopause	11,311 (56.3)	11,396 (56.5)	11,712 (58.0)	12,493 (61.0)	13,546 (66.2)	<0.0001
Pre-menopause	8765 (43.7)	8787 (43.5)	8487 (42.0)	7994 (39.0)	6928 (33.8)	
Family history of cancer						
Yes	8778 (26.8)	9157 (28.0)	8980 (27.4)	8882 (27.1)	8609 (26.3)	<0.0001
No	23,979 (73.2)	23,564 (72.0)	23,777 (72.6)	23,851 (72.9)	24,083 (73.7)	

^a^. Quintile 1 indicates least inflammatory diet (lowest DII), and Quintile 5 indicates most inflammatory diet (highest DII). ^b^. The data of continuous variables are presented as mean with standard deviation, and the data of categorical variables are presented as frequency number with percentage. ^c^. *p* values for trends were calculated using the Jonckheere–Terpstra test for continuous variables and the Mantel–Haenszel Chi-square test for categorical variables. ^d^. Regularity of physical activity was defined according to whether or not subjects participated regularly in any sports to the point of sweating.

**Table 2:** Cox proportional Hazard Ratios (HRs) with 95% Confidence Intervals (CIs) for incidence of cancer expression according to Dietary Inflammatory Index (DII), KoGES 2004–2013.

	Quintiles of Dietary Inflammatory Index (DII) ^a^					*p* Value ^b^	*p* Value ^c^
	Q1	Q2	Q3	Q4	Q5		
All subjects							
Person-years	255,579	245,501	243,536	240,475	230,054		
Incidence (*n*)	353	317	354	330	289		
Unadjusted	1.00	0.94 (0.81–1.10)	1.05 (0.91–1.22)	1.04 (0.89–1.20)	1.14 (0.98–1.34)	0.05	0.11
Fully adjusted ^d^	1.00	0.99 (0.84–1.16)	1.10 (0.93–1.29)	1.11 (0.93–1.32)	1.20 (0.99–1.45)	0.03	0.0432
Male							
Person-years	83,738	84,350	85,104	82,214	82,916		
Incidence (N)	118	91	108	102	101		
Unadjusted	1.00	0.73 (0.56–0.96)	0.82 (0.63–1.06)	0.84 (0.64–1.09)	0.98 (0.75–1.27)	0.89	0.67
Fully adjusted ^d^	1.00	0.69 (0.52–0.92)	0.74 (0.55–1.00)	0.75 (0.55–1.03)	0.80 (0.58–1.10)	0.42	0.16
Female							
Person-years	171,841	161,151	158,432	158,261	147,138		
Incidence (N)	235	226	246	228	188		
Unadjusted	1.00	1.06 (0.88–1.27)	1.18 (0.99–1.41)	1.14 (0.9–1.37)	1.23 (1.0–1.49)	<0.0001	<0.0001
Fully adjusted ^d^	1.00	1.16 (0.96–1.41)	1.31 (1.07–1.61)	1.32 (1.06–1.64)	1.44 (1.14–1.82)	0.002	0.0006

^a^. Quintile 1 indicates least inflammatory diet (lowest DII), and Quintile 5 indicates most inflammatory diet (highest DII); Q1 is reference (HR=1.00). ^b^. *p* values for trend were calculated with categorical DII values. ^c^. *p* values for trend were calculated with continuous DII values. ^d^. Adjusted model regarded continuous variables for age and energy and categorical variables for gender, marital status, education level, smoking, drinking, physical activity, and family history of cancer. * *p* interaction for gender and categorical DII in fully adjusted Cox model = 0.0594.

**Table 3:** Multivariable Cox proportional Hazard Ratios (HRs) with 95% Confidence Intervals (CIs) for components effecting reduced DII level in women.

Components	Quintiles of DII Components ^a^					*p* Value ^b^	*p* Value ^c^
	Q1	Q2	Q3	Q4	Q5		
Isoflavone							
Incidence (*n*)	197	224	252	223	227		
HRs ^d^	1.00	0.99 (0.81–1.20)	1.06 (0.88–1.28)	0.87 (0.72–1.06)	0.78 (0.64–0.95)	0.003	0.005
Flavanone							
Incidence (*n*)	193	167	199	267	297		
HRs ^d^	1.00	0.78 (0.63–0.97)	0.85 (0.69–1.04)	0.97 (0.80–1.17)	0.81 (0.67–0.98)	0.31	0.16
Flavonol							
Incidence (*n*)	167	237	251	254	214		
HRs ^d^	1.00	1.05 (0.86–1.29)	0.89 (0.73–1.09)	0.79 (0.65–0.97)	0.66 (0.54–0.82)	<0.0001	0.002
Flavan-3-ol							
Incidence (*n*)	165	260	243	244	211		
HRs ^d^	1.00	1.06 (0.86–1.29)	0.93 (0.76–1.14)	0.81 (0.66–0.99)	0.68 (0.55–0.84)	<0.0001	0.002
Green and Black tea							
Incidence (*n*)	412	12	242	246	211		
HRs ^d^	1.00	0.59 (0.32–1.09)	0.89 (0.76–1.05)	0.78 (0.66–0.91)	0.65 (0.54–0.77)	<0.0001	0.002
Riboflavin							
Incidence (*n*)	200	220	225	246	232		
HRs ^d^	1.00	1.05 (0.86–1.28)	1.06 (0.87–1.29)	1.04 (0.86–1.26)	0.81 (0.67–0.99)	0.03	0.05
Fe							
Incidence (N)	203	203	225	251	241		
HRs ^d^	1.00	0.94 (0.77–1.14)	0.97 (0.80–1.18)	0.99 (0.82–1.20)	0.80 (0.66–0.97)	0.05	0.03

^a^. Quintile 1 has the least amount of DII components, and Quintile 5 has the most amount of DII components; Q1 is reference (HR = 1.00). ^b^. *p* values for trends were calculated with categorical components. ^c^. *p* values for trends were calculated with continuous components. ^d^. Each of the components are adjusted for age and energy as continuous variables and gender, marital status, education level, smoking, drinking, physical activity, and family history of cancer as categorical variables.
